# Retraction: The oncolytic virus in cancer diagnosis and treatment

**DOI:** 10.3389/fonc.2023.1320477

**Published:** 2023-11-10

**Authors:** Frontiers Editorial Office

Following publication, concerns were raised regarding the scientific validity of the article. An investigation was conducted in accordance with Frontiers’ policies. It was found that these concerns were valid and that the article does not meet the standards of editorial and scientific soundness for Frontiers in Oncology; therefore, the article has been retracted.

This retraction was approved by the Chief Editors of Frontiers in Oncology and the Chief Executive Editor of Frontiers. The authors do not agree to this retraction.

